# Multimodal treatment and long-term survival in a rare case of melanoma with pancreatic and splenic metastases: a case report and literature review

**DOI:** 10.3389/fonc.2026.1736458

**Published:** 2026-06-10

**Authors:** Yanhua Mou, Lan Han, Jing Yu, Hui Shen, Hui Li, Lichao Xu, Qingfeng Li

**Affiliations:** 1 Department of Oncology, Xiangyang Central Hospital, Affiliated Hospital of Hubei University of Arts and Science, Xiangyang, Hubei, China; 2 Institute of Oncology, Hubei University of Arts and Science, Xiangyang, Hubei, China; 3 Department of Pathology, Xiangyang Central Hospital, Affiliated Hospital of Hubei University of Arts and Science, Xiangyang, Hubei, China

**Keywords:** BRAF V600 mutation, melanoma, pancreatic metastasis, targeted therapy, toripalimab

## Abstract

**Background:**

Patients with melanoma frequently develop metastases, and managing these cases, especially aggressive, rare types such as pancreatic metastasis, remains challenging. To date, no consensus on treatment has been reached.

**Case presentation:**

This study presents an uncommon case of a 55-year-old man with rapidly progressive metastatic melanoma following initial lower-extremity melanoma resection. Within 5 months postoperatively, he developed widespread metastases, including lesions in the chest, back, calves, axilla, pancreas, and spleen, an exceptionally rare presentation. The patient received various treatment regimens, including toripalimab monotherapy, combination therapy with anlotinib, and combination therapy with temozolomide. Ultimately, surgical resection of the pancreatic and spleen metastases revealed a *BRAF* V600 mutation, permitting successful transition to targeted therapy with dabrafenib plus trametinib. As of the most recent follow-up on March 6, 2025, the patient remains stable with long-term survival.

**Conclusion:**

This case report, along with the subsequent literature review, emphasizes the need for continued treatment and vigilance of molecular monitoring in patients with advanced melanoma, as well as the critical role of multidisciplinary team collaboration in managing complex metastases. Surgical resection and targeted therapy might yield favorable outcomes for select patients with melanoma and pancreatic and splenic metastases.

## Introduction

Melanoma is an aggressive malignancy of melanocytes derived from the neural crest (1). As the third most common and deadliest form of skin cancer, melanoma poses a clinical challenge because of its high metastatic potential (2, 3). Alarmingly, its incidence has continued to rise in recent decades. Ultraviolet radiation is a major risk factor for melanoma, and effective sun protection measures can reduce its occurrence. Cutaneous melanoma, which accounts for over 90% of cases, is classified into four major histological subtypes: superficial spreading, nodular, lentigo maligna, and acral lentiginous melanoma, each exhibiting distinct clinical and pathological features. Early diagnosis is crucial for improving patient outcomes. In clinical practice, the ABCDE criteria (Asymmetry, Border, Color, Diameter, Evolution) are utilized for initial screening, while dermoscopy enhances diagnostic accuracy for early or atypical lesions. However, definitive diagnosis remains dependent on histopathological examination (4). Over the past decade, the emergence of targeted therapy and immunotherapy has revolutionized the treatment landscape for melanoma (5). For instance, the combination of BRAF and MEK inhibitors in patients with *BRAF* V600 mutations has achieved high overall response rates (ORRs) ranging from 63% to 70%, along with a 5-year overall survival (OS) rate of 34% (6–12). In the realm of immunotherapy, the 5-year OS rate for melanoma patients treated with nivolumab and ipilimumab exceeds 50% (13).

Melanoma has a high potential to metastasize to multiple organs, with distant metastases commonly involving the brain, lungs, and liver. Notably, approximately 50% of patients with melanoma present with melanoma brain metastasis (MBM) (14). In contrast, metastasis to the pancreatic or spleen from melanoma is rare, occurring in fewer than 2% of cases (15, 16). The optimal management of melanoma with pancreatic or spleen metastasis remains controversial, and no consensus has been reached. Therefore, further studies are needed to refine treatment strategies for melanoma with pancreatic or spleen metastasis.

This study reports a rare case of widely metastatic melanoma. Through a multimodal treatment approach involving immunotherapy, targeted therapy, chemotherapy, and surgical resection, the patient achieved long-term survival. The treatment course highlights key aspects, including the selection and sequencing of immunotherapy and targeted therapy, the potential pseudoprogression (PsP) and vessel co-option following the combination of immunotherapy with anti-angiogenic therapy, and the surgical management of rare metastatic sites. We also conducted a systematic review of the relevant literature.

## Case presentation

**Figure 1:**
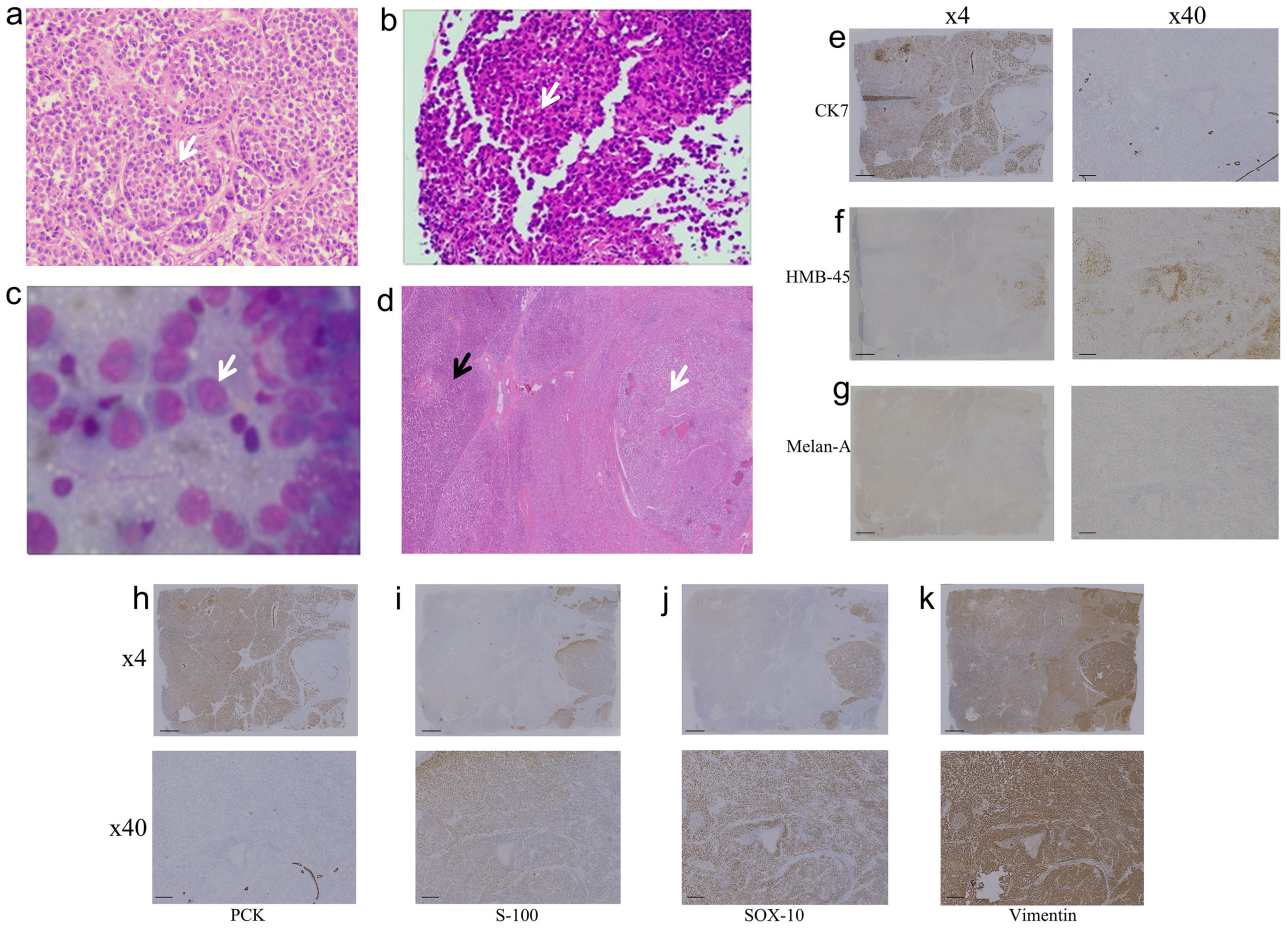
Histopathological and cytological findings in the patient. **(a)** Initial surgical specimen (left thigh tissue) confirmed as melanoma (histopathology) in September 2019 (×10). **(b)** Biopsy of the back lesion and mass in March 2020 revealed disease progression: metastatic melanoma (histopathology) (×10). **(c)** Cytological analysis of a newly formed mass on the left back in July 2020: metastatic melanoma (×100). **(d)** Histopathological examination of the pancreatic metastasis resected in March 2023 confirmed metastatic melanoma (×10). White arrows indicate the tumor, and black arrows indicate the pancreas. (**e–k**) Immunohistochemical staining of pancreatic tumor tissue revealed the following results: S-100 (+), HMB-45 (partially +), Melan-A (–), SOX-10 (+), VIM (+), CK7 (–), and PCK (–). The images show views at ×4 magnification (scale bar: 2.0mm) and at ×40 magnification (scale bar: 300µm).

A 55-year-old man visited our hospital on March 16, 2020, because of a 1-week history of left lower-limb pain. Six to seven months prior, he had noticed a subcutaneous mass on his right thigh over the course of 1 month, and he underwent surgical resection of the mass at an external hospital on September 18, 2019. Postoperative pathological examination confirmed a diagnosis of melanoma (**Figure 1a**). No adjuvant treatment was administered postoperatively.

**Figure 2:**
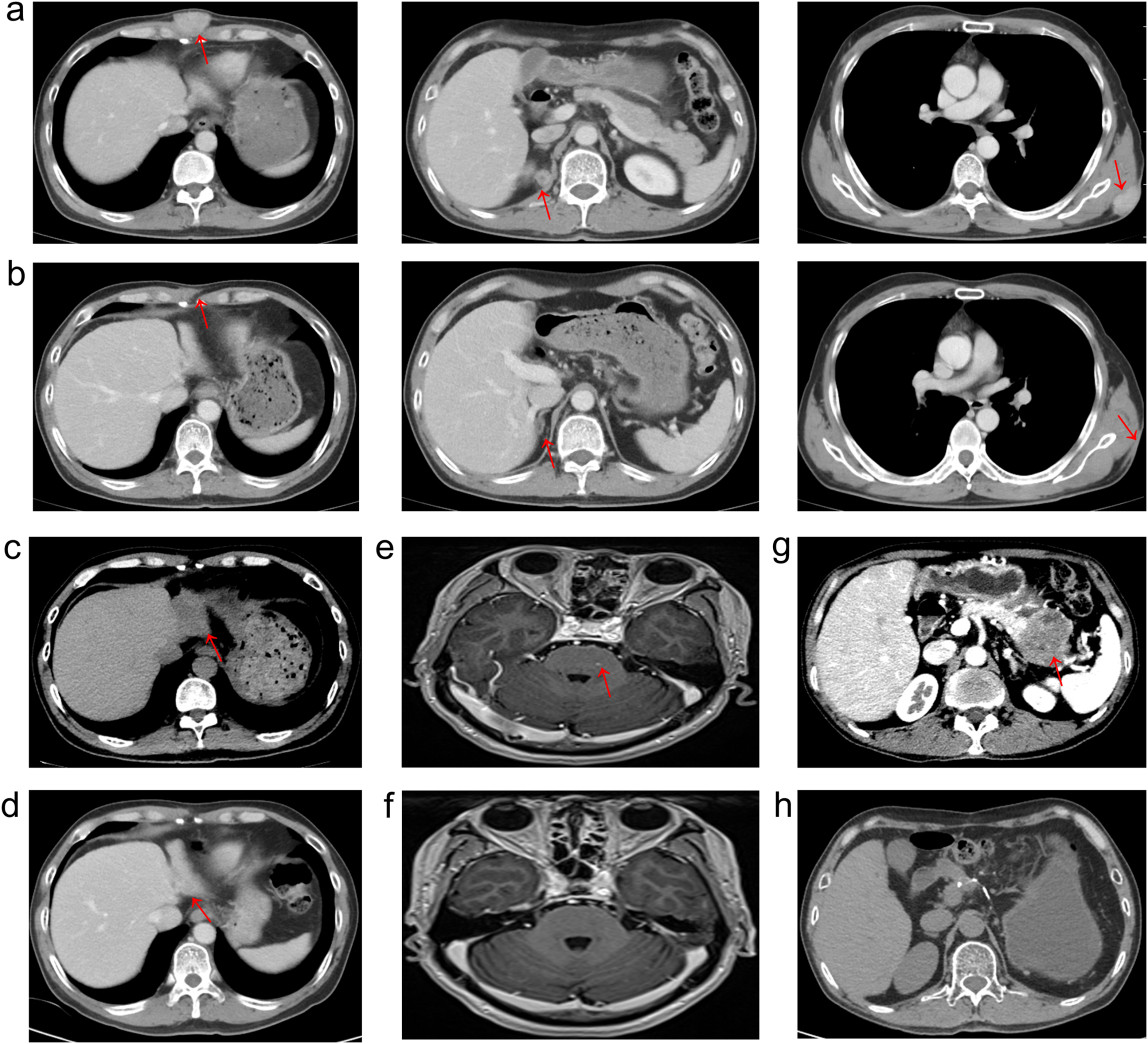
Imaging findings in the patient. **(a)** After the initial surgery, the lesion recurred, manifesting as subcutaneous masses located below the xiphoid process, near the edge of the right kidney, and on the left side of the back [computed tomography (CT)]. **(b)** Following toripalimab immunotherapy, the masses under the xiphoid process, adjacent to the right kidney, and in the back had decreased in size (CT). **(c)** After combined treatment with toripalimab and anlotinib, follow-up CT in September 2020 revealed a soft-tissue mass in the hepatogastric space. **(d)** Upon discontinuation of anlotinib and continuation of toripalimab, follow-up CT demonstrated regression of the soft-tissue mass in the hepatogastric space. **(e)** Abnormal hyperintense foci and an enhancing lesion in the left pons, suggestive of metastasis [cranial magnetic resonance imaging (MRI)]. **(f)** After chemotherapy and immunotherapy, the pontine enhancing focus was resolved (MRI). **(g)** A soft-tissue mass in the pancreatic tail, suggestive of pancreatic metastasis (positron emission tomography-CT). **(h)** The latest follow-up imaging indicated stable disease. Red arrows indicate the tumor location.

Subsequent computed tomography (CT) revealed multiple soft-tissue density nodules and masses of varying sizes in both axillary regions, as well as subcutaneous areas of the chest and back, near the right renal margin, and along the right abdominal wall (**Figure 2a**). Magnetic resonance imaging (MRI) identified small nodules within the right gluteus maximus and soleus muscles of the left calf, accompanied by perilesional muscle edema and multiple enlarged LNs in both inguinal regions, all suggestive of metastases from melanoma. A biopsy of a subcutaneous mass beneath the back and below the xiphoid process confirmed the diagnosis of metastatic melanoma (**Figure 1b**).

Due to financial constraints and personal reluctance, the patient declined further genetic testing and systemic chemotherapy, opting instead to initiate treatment with toripalimab (210 mg, every 2 weeks) on March 18, 2020. Follow-up CT demonstrated a reduction in the size of all lesions (**Figure 2b**).

On July 17, 2020, the patient developed new masses in the left shoulder, back, and forearm. Cytological examination of the back mass confirmed the presence of metastatic melanoma (**Figure 1c**). CT revealed multiple soft-tissue nodules and masses of varying sizes in both axillary regions, subcutaneous areas of the chest and back, near the right renal margin, and along the right abdominal wall. Multiple enlarged LNs in both inguinal regions exhibited partial resolution or reduction. On July 18, 2020, the patient began combination therapy with toripalimab (210 mg every 2 weeks) and anlotinib (12 mg once daily for 14 days in each 3-week cycle). On September 9, 2020, the patient reported abdominal pain. Follow-up CT identified an enlarged soft-tissue mass (5.1 × 3.5 cm^2^) in the hepatogastric space with ill-defined boundaries adjacent to the gastric antrum and left hepatic lobe (**Figure 2c**), whereas the residual metastases had slightly decreased in size. The patient subsequently opted for monotherapy with toripalimab (210 mg every 2 weeks). On November 5, 2020, follow-up imaging identified a reduction in the hepatogastric mass (4.9 × 1.7 cm^2^; **Figure 2d**), partial regression of multiple axillary LNs, and stable disease. Cranial MRI detected abnormal hyperintense foci in the left pons (**Figure 2e**), suggesting metastatic involvement. The patient refused to receive radiotherapy (RT) for the brain lesions, and the treatment was adjusted to toripalimab (210 mg every 2 weeks) combined with temozolomide (360 mg orally administered on days 1–5 of each 4-week cycle). After two cycles of treatment, the brain lesions resolved (**Figure 2f**), whereas other metastases remained stable. The patient then resumed toripalimab monotherapy (210 mg every 2 weeks), maintaining stable disease throughout treatment.

On March 7, 2023, the patient’s condition featured significant new change. He reported intermittent abdominal pain and had an ECOG performance status of 1. Positron emission tomography-CT revealed the presence of soft-tissue masses in the body, tail, and anterior neck of the pancreas (**Figure 2g**), and these lesions were characterized by elevated glucose metabolism. The larger lesions were accompanied by splenic vein tumor thrombus, suggesting malignant tumors with a high potential for metastasis.

Following multidisciplinary consultation, a laparoscopic procedure was performed on March 14, 2023, including resection of the pancreatic body and tail, splenectomy, resection of the transverse mesocolon tumor, and regional LN dissection. Postoperative pathology confirmed metastatic melanoma involving the pancreatic body and tail (**Figure 1d**), spleen, and mesenteric nodules. Tumor invasion was observed in the pancreatic and splenic portal connective tissues, with neuroinvasion noted in biopsy specimens. No evidence of tumor spread was detected in the surrounding LNs. Immunohistochemical staining revealed the expression status of the following markers: S-100 (+), HMB-45 (partially+), Melan-A (–), SOX-10 (+), VIM (+), CK7 (–), and PCK (–) (**Figures 1e–k**).

**Figure 3:**
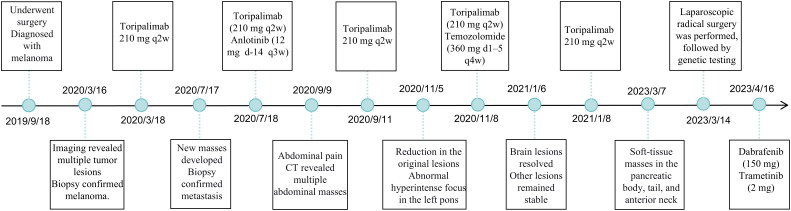
Timeline of diagnosis, treatment, genetic testing, and follow−up.

Genetic testing utilizing next-generation sequencing was conducted at the Department of our hospital on surgically resected pancreatic metastatic tissue, which identified the *BRAF* V600E mutation. In late April 2023, the patient was treated with a combination of dabrafenib (150 mg po twice daily) and trametinib (2 mg po once daily). During the latest follow-up on March 6, 2025 (**Figure 3**), the patient exhibited stable disease (**Figure 2h**) without significant symptoms with an Eastern Cooperative Oncology Group performance status of 0.

## Discussion

Melanoma is highly malignant because of its aggressive biological behavior, frequently metastasizing to LNs and vital organs (17). This case study presents a case of melanoma with extensive subcutaneous, brain, mesenteric, pancreatic, and splenic metastases. Long-term survival was achieved after multimodal therapy, including immunotherapy, targeted therapy, chemotherapy, and surgery.

Brain metastases is a common site of metastasis for melanoma tumors (18–21). Currently, there is no consensus on the standard treatment for MBM. Preferred options include local therapies, specifically surgical resection and/or RT (SRS and WBRT). Temozolomide, an orally administered alkylating agent capable of crossing the BBB, has demonstrated clinical responses in some patients with MBM. Furthermore, recent advancements in systemic therapies, such as immune checkpoint inhibitors (ICIs) and BRAF plus MEK inhibitors, have opened new avenues for treating MBM. In this case, we used immunotherapy combined with chemotherapy to treat brain metastases, resulting in favorable outcomes. This suggests that, for select patients with brain metastases, the combination of immunotherapy and chemotherapy could serve as an effective salvage therapy.

Metastasis of melanoma to the pancreas is relatively uncommon (16). Currently, there is no consensus on treating pancreatic metastases from melanoma. With advancements in pancreatic surgery, surgical resection has emerged as an effective therapeutic approach, with median survival improving from 5–8 months to 15–28 months, including documented long-term survivors (22). In a study involving 60 patients with melanoma and gastrointestinal metastases, eight patients presented with pancreatic metastasis. Of these, six patients underwent curative surgical resection, achieving an impressive 5-year survival rate of 50% (23). Deutsch et al. reported significantly better OS in patients with melanoma and pancreatic metastases undergoing surgical intervention than in those receiving non-surgical treatment (18 months vs. 7 months) (24). Reddy and Wolfgang reported surgical outcomes for pancreatic metastasectomy in 11 patients with metastatic melanoma, demonstrating a median survival of 14 months (25). The indications for pancreatic metastasectomy are currently limited to patients with good performance status, well-controlled primary disease, and radiologically confirmed resectable tumors. However, because of the highly aggressive nature of melanoma, surgical intervention alone might be insufficient for disease control. A multimodal approach incorporating systemic therapies, such as chemotherapy, immunotherapy, and targeted therapy, is essential for improving treatment efficacy. Consequently, therapeutic decisions should involve multidisciplinary team (MDT) discussions, considering surgical feasibility, molecular characteristics, and response to systemic therapies to achieve personalized precision medicine. In the current case, the patient underwent pancreatic metastasectomy following MDT discussion and subsequently received sequential targeted therapy.

Splenic metastasis from solid tumors is relatively uncommon, likely owing to the lack of afferent lymphatics in the spleen (26–30). Patel et al. reported that splenic metastasis from malignant melanoma occurred in 3.2% of cases, all of which were accompanied by metastases to other organs (liver, lungs, and bones) (31). Isolated splenic metastases are particularly rare. Wilt et al. found that the median OS for patients with melanoma splenic metastasis after splenectomy was 11 months, with a subgroup of patients with isolated metastasis displaying superior survival of 23 months and two patients remaining disease-free for more than 2 years during follow-up. By contrast, conservatively managed patients exhibited a significantly shorter median OS of only 4 months (32). Wood et al. analyzed patients with melanoma undergoing adrenalectomy, hepatectomy, splenectomy, or pancreatectomy and noted that those who underwent complete resection had significantly better median OS than those who underwent incomplete resection (23). Interestingly, there was no significant difference in median OS between patients with single metastases and those with synchronous multiple metastases. Therefore, in highly selected patients with melanoma and intra-abdominal solid organ metastases, aggressive pursuit of complete surgical resection might prolong OS. In the present case, the patient presented with multifocal intra-abdominal metastases involving the pancreas, spleen, and mesentery, while maintaining good extra-abdominal disease control, leading to the decision for radical abdominal surgery.

Melanoma exhibits remarkable diversity in terms of driver mutations and common genetic alterations. Different melanoma subtypes exhibit distinct driver genetic changes, particularly in the mitogen-activated protein kinase pathway, a crucial pathway for cellular proliferation that frequently is constitutively activated in melanomas. The BRAF oncogene is the most commonly altered gene in this pathway, with mutations occurring in approximately 40%–50% of all patients with melanoma (5, 33, 34). The predominant mutation, V600E, accounts for 70%–90% of *BRAF* mutations, followed by V600K, V600D, and V600R (9, 35–37). Clinical trials have confirmed that combination therapy with BRAF inhibitors and MEK inhibitors offers significant survival benefits for BRAF V600-mutant melanoma. Multiple phase III studies have demonstrated that various dual-agent combination regimens (such as vemurafenib plus cobimetinib, dabrafenib plus trametinib, and encorafenib plus binimetinib) provide clear clinical benefits for patients (8, 12, 38). In this case, the melanoma patient harbored a BRAF V600 mutation. We administered an antitumor regimen of trametinib in combination with dabrafenib, and the patient achieved a very favorable response. Moreover, during the course of dual-targeted therapy, no significant adverse effects were observed.

BRAF inhibitor/MEK inhibitor combination therapy significantly extends survival, but resistance develops within 11–15 months. Preclinical studies demonstrated that BRAF inhibitor/MEK inhibitor therapy can enhance tumor susceptibility to ICIs through microenvironment modulation, suggesting that combining targeted agents with immunotherapy is a promising treatment approach. Multiple studies have reported that in patients with BRAF V600 mutant melanoma, the triplet regimen of PD-1/PD-L1 inhibitors combined with dual-targeted therapy (BRAF inhibitor plus MEK inhibitor) significantly prolongs PFS and duration of response compared with dual-targeted therapy alone (39). However, triple therapy is associated with a higher incidence of adverse events, prompting some studies to explore the sequence or alternatives to optimize efficacy while minimizing toxicity. Some studies found that administering immunotherapy followed by targeted therapy, especially anti-PD-1/anti-CTLA-4 therapy followed by BRAF inhibitor/MEK inhibitor regimens, is more effective (40). However, a retrospective analysis suggested that anti-PD-1 therapy can negatively affect subsequent BRAF inhibitor therapy, as patients developed drug resistance after immunotherapy (41–43). In this case, the patient initially received immunotherapy and was subsequently switched to targeted therapy after disease progression, achieving long-term survival. Immunotherapy generally does not induce resistance mutations in the BRAF pathway. In contrast, resistance to BRAF/MEK inhibitors may lead to immunosuppressive changes in the tumor microenvironment, thereby reducing sensitivity to subsequent immunotherapy. Therefore, for patients with BRAF V600 mutant melanoma, a sequential therapy approach that combines immunotherapy followed by targeted therapy may represent the optimal treatment strategy.

Immunotherapy has revolutionized the treatment landscape for various cancers, including melanoma, one of the first malignant tumors to be treated with this approach (44). High-dose interleukin-2 was the first immunotherapy approved for melanoma; however, it frequently caused severe toxicities and failed to improve survival (45, 46). In recent years, CTLA4 and PD1 antibody immunotherapies have significantly improved survival rates in patients with metastatic melanoma. Ipilimumab, a CTLA-4 antibody, was the first immune checkpoint inhibitor to demonstrate a survival benefit in melanoma (47, 48). Subsequently, the PD-1 inhibitor pembrolizumab was shown to be more efficacious compared to ipilimumab (49, 50). Moreover, the combination of nivolumb (PD-1 inhibitor) and ipilimumab yielded a longer median PFS compared with either agent alone (51, 52). Consequently, anti-PD-1 therapy, either alone or in combination with anti-CTLA-4 therapy, is currently recommended for the treatment of advanced melanoma. In this case, toripalimab (PD-1 antibody) monotherapy was chosen because of the patient’s financial limitations and drug accessibility, resulting in a favorable clinical benefit.

However, the advent of immunotherapy has attracted attention to PsP, first noted in patients with melanoma treated with ipilimumab. A comprehensive review of PsP across different tumor types indicates that the highest incidence of PsP following ICIs therapy occurs in patients with melanoma, ranging from 4.6% to 9.3%. PsP is characterized by tumor enlargement or new lesions without clinical deterioration, followed by tumor shrinkage or disease stabilization during immunotherapy (53, 54). At present, histopathological examination is the gold standard for confirming PsP. Accurate assessment of PsP presents a significant challenge for clinicians. In this case, the patient developed new lesions following treatment with immunotherapy combined with anlotinib, and these lesions subsequently shrank after switching to immunotherapy alone, which is consistent with the characteristics of pseudoprogression. Unfortunately, a biopsy of the new lesions was not performed to confirm the diagnosis. An alternative explanation for this specific scenario is the vessel co−option mechanism induced by anlotinib, whereby the new lesions resulted from the “escape” of certain tumor cells through vessel co−option. This case suggests that new lesions emerging during combined immunotherapy and anti−angiogenic therapy do not necessarily indicate true disease progression. Both PsP and resistance mediated by vessel co−option can manifest as radiological “progression, “ and either may be managed with the continuation or adjustment of the treatment regimen. In such situations, dynamic imaging follow−up, multidisciplinary collaboration, and pathological biopsy when necessary are essential to avoid prematurely abandoning effective treatment.

## Conclusions

In conclusion, this study presents a rare case of metastatic melanoma in a patient who received sequential treatments, including toripalimab monotherapy, combination therapy with anlotinib, and chemotherapy. Following the surgical resection of pancreatic and splenic metastases, a *BRAF* V600 mutation was identified, enabling a transition to targeted therapy, contributing to long-term survival. Collectively, this case study, together with those in the literature, underscores the necessity for ongoing treatment and vigilance in molecular monitoring for advanced melanoma. Immunotherapy followed by sequential targeted therapy may represent the optimal approach, and clinicians should remain alert for PsP and vessel co-option following combined immunotherapy and anti-angiogenic therapy, as well as the critical role of MDT collaboration in managing complex cases of metastasis. Furthermore, surgical resection may offer favorable outcomes for select melanoma patients with pancreatic and splenic metastases.

## Data Availability

The original contributions presented in the study are included in the article/supplementary material. Further inquiries can be directed to the corresponding authors.
